# Enteroaggregative *Escherichia coli* Related to Uropathogenic Clonal Group A

**DOI:** 10.3201/eid1305.061057

**Published:** 2007-05

**Authors:** Faith Wallace-Gadsden, James R. Johnson, John Wain, Iruka N. Okeke

**Affiliations:** * Haverford College, Haverford, Pennsylvania, USA;; † Minneapolis Veterans Administration Medical Center, Minneapolis, Minnesota, USA;; ‡ University of Minnesota, Minneapolis, Minnesota, USA; and; § Wellcome Trust Sanger Institute, Cambridgeshire, United Kingdom; 1 Current affiliation: Tufts University School of Medicine, Boston, Massachusetts, USA

**Keywords:** enteroaggregative Escherichia coli, clonal group A, antimicrobial resistance, antibiotic resistance, multilocus sequence typing, E. coli infections, dispatch

## Abstract

Enteroaggregative *Escherichia coli* (EAEC) are heterogeneous, diarrheagenic *E. coli.* Of EAEC strains from Nigeria, 10 independent antimicrobial-resistant isolates belonged to the multilocus sequence type 69 clonal complex, to which uropathogenic *E. coli* clonal group A belongs. This finding suggests a recent common ancestor for these distinct groups of pathogenic *E. coli.*

Enteroaggregative *Escherichia coli* (EAEC) is an emerging category of diarrheagenic *E. coli.* EAEC are heterogeneous, and the distribution of known virulence genes rarely correlates with phylogeny based on housekeeping loci (*1*). We recently identified 2 loci, involved in iron acquisition, that are distributed among EAEC in a manner that correlates with multilocus enzyme electrophoresis typing based on 20 housekeeping enzymes (*2*). This finding supports the possibility that EAEC clonal groups with increased pathogenic potential exist.

## The Study

To identify overrepresented subgroups of potential clonal origin, we examined 131 EAEC strains isolated from children in Nigeria (*3*), 73 from 187 children with diarrhea and 58 from 144 healthy control participants. All 131 strains had previously been categorized as EAEC by the standard HEp-2 cell adherence assay (*3**,**4*). To determine flagellin types, we used an *Rsa*I-based PCR–restriction fragment length polymorphism (RFLP) protocol. Using primers F-FLIC1 (5′-ATGGCACAAGTCATTAATACCCAAC-3′) and R-FLIC2 (5′-CTAACCCTGCAGCAGAGACA-3′), we obtained an internal *fliC* amplicon from 105 (80.1%) of the 131 isolates. RFLP analysis delineated ≥31 flagellin genotypes among these amplicons (Appendix Table). Using 16 control strains, which represented 12 antigenically distinct H-types verified at reference centers, we could associate a specific H antigen with 10 of these RFLP patterns. The most common pattern, which corresponded with the H18 antigen, accounted for 18 (17%) of the genotyped isolates (or 14% of all isolates). Two H7 control strains had different genotypes, which indicates that the products of 2 different alleles are recognized by anti-H7 antiserum. Both genotypes were detected among the test EAEC strains (Appendix Table) and together accounted for 10 (7.6%) of the isolates. Other predominant *fliC* variants were H2 (3%), H11 (3.8%), H21 (7.6%), and H45 (4.9%). Although some *fliC* variants were somewhat more common among strains from children with diarrhea than from control participants (Appendix Table), differences were not statistically significant (p>0.05, Fisher exact test).

Antimicrobial susceptibility profiles were determined by disk diffusion as specified by the Clinical and Laboratory Standards Institute (*5*). Disks containing ampicillin (10 µg), tetracycline (30 µg), trimethoprim (5 µg), nalidixic acid (30 µg), chloramphenicol (30 µg), sulfonamide (300 µg), streptomycin (10 µg), and ciprofloxacin (5 µg) were used for testing on Mueller-Hinton agar (Oxoid, Lenexa, Kansas, USA). Of the 18 Nigerian H18 strains, 13 had the same resistance pattern: ampicillin, chloramphenicol, streptomycin, sulfonamide, tetracycline, trimethoprim (Table 1). PCR identified 3 EAEC-specific and 2 other virulence genes (*2**,**6*). Markers of well-characterized aggregative adherence plasmids, present in typical EAEC strains, are aggregative adherence regulator gene *aggR*, anti-aggregative protein or dispersin gene *aap,* and empiric plasmid probe (CVD432) that represents part of the *aat* secretion system operon (*6*). Of the 131 isolates, <30% harbor these loci (*7*). However, 17 (89%) of 18 H18-positive EAEC isolates harbored ≥1 of these aggregative adherence plasmid loci, and 15 (83%) of 17 harbored all 3 loci (Table 1). Moreover, 15 (83%) of the 18 isolates contained *iucA* (aerobactin synthesis), and 17 (94%) contained *chuA* (heme transport outer-membrane receptor), significantly more than the other 113 EAEC isolates (and 39.8% and 24.8% for *iucA* and *chuA,* respectively; p<0.001 for each).

**Table 1:** Properties of H18 EAEC isolates and selected reference strains*

Strain	Country of isolation	Clinical condition	Serotype	Motility	CGA (*fumC* SNP)	Resistance pattern	*aggR*	*aap*	CVD432 (*aat)*	*astA*	*chuA*	*iucA*
C08	Nigeria	Diarrhea	O86:H18	+	+	Ap St Su Tc Tp	+	+	+	–	+	+
C14	Nigeria	Diarrhea	N/K	+	+	Ap Cm St Su Tc Tp	+	+	+	+	+	+
E23	Nigeria	Diarrhea	N/K	+	+	Ap Cm St Su Tc Tp	+	+	+	–	+	+
E30	Nigeria	Diarrhea	N/K	+	+	Ap Cm St Su Tc Tp	+	+	+	–	+	+
G10	Nigeria	Diarrhea	N/K	+	+	Ap Cm St Su Tp	+	+	+	–	+	+
G17a	Nigeria	Diarrhea	N/K	–	+	Ap Cm St Su Tc Tp	+	+	+	–	+	+
G59	Nigeria	Diarrhea	N/K	–	+	Ap Cm St Su Tc Tp	+	+	+	–	+	+
G67b	Nigeria	Diarrhea	N/K	+	+	Ap Cm St Su Tc Tp	+	+	+	–	+	+
C16	Nigeria	Diarrhea	N/K	+	–	Ap Cm St Su Tc Tp	+	+	+	–	+	–
G55	Nigeria	Diarrhea	N/K	+	–	Ap Cm St Su Tc Tp	–	–	–	–	–	–
E64	Nigeria	Healthy	N/K	+	+	Ap Cm St Su Tc Tp	+	+	+	+	+	+
G108	Nigeria	Healthy	N/K	+	+	Ap Cm St Su Tc Tp	–	–	+	+	+	+
E56	Nigeria	Healthy	N/K	+	–	Ap Cm St Su Tc	+	+	+	–	+	+
E62	Nigeria	Healthy	N/K	–	–	Tc	+	+	+	–	+	+
E68	Nigeria	Healthy	N/K	+	–	Ap Cm St Su Tc Tp	–	–	+	+	+	+
G103	Nigeria	Healthy	N/K	+	–	Ap Cm St Su Tc Tp	–	–	+	+	+	+
G121a	Nigeria	Healthy	N/K	+	–	Ap Cm St Su Tc Tp	+	+	+	–	+	+
G149	Nigeria	Healthy	N/K	+	–	Ap St Su Tc Tp	+	+	+	–	+	+
O42	Peru	Diarrhea	O44:H18	+	–	Cm St Su Tc Tp	+	+	+	–	+	–
44-1	Thailand	Diarrhea	O36:H18	+	–	Ap Cm St Su Tc	+	+	+	–	+	+
144-1	Thailand	Diarrhea	O77:NM	–	+	Cm	+	+	+	–	+	+
E02	Nigeria	Diarrhea	Ont:H18	+	–	Ap Cm St Su Tc Tp	–	–	–	–	+	–
DH5α†	N/A	N/A	N/A	+	–	–	–	–	–	–	–	–
2P9‡	USA	UTI	O15:K52:H1	ND	–	St	–	–	–	–	+	+
SEQ102‡	USA	UTI	O11:NT	ND	+	Ap Cm St Su Tc Tp	–	–	–	–	+	+
UMN026‡	USA	UTI	O17:K52:H18	ND	+	Ap Cm St Tc Tp	–	–	–	–	+	+
CFT073‡	USA	UTI	O6:K2:H1	ND	–	–	–	–	–	–	+	+

*EAEC, enteroaggregative Escherichia coli; CGA, clonal group A; Ap, ampicillin; St, streptomycin; Su, sulfonamide; Tc, tetracycline; Tp, trimethoprim; N/K, not known; Cm, chloramphenicol; ETEC, enterotoxigenic E. coli; N/A, not applicable; UTI, urinary tract infection. All strains were susceptible to nalidixic acid and ciprofloxacin. All pathotypes are EAEC (H18) unless otherwise noted. †Pathotype K-12. ‡Pathotype uropathogenic *E. coli*.

Multidrug-resistant, *chuA*-positive *E. coli* H18 strains are also frequently recovered from patients with urinary tract infection. Some of these strains derive from the successful and globally disseminated multidrug-resistant clonal group A (CGA) (*8*). Uropathogenic *E. coli* (UPEC) clonal group A strains occur in the United States and Europe; typically exhibit serotypes O11:H18, O17:H18, O73:H18, or O77:H18; and share a common resistance and repetitive element. PCR profile (*8*). Recently, at certain US centers, ≥33% of trimethoprim-resistant *E. coli* isolates from uncomplicated cases of pyelonephritis and cystitis have represented CGA (*9*). CGA strains also can infect nonurinary, extraintestinal sites (*10*). Furthermore, CGA-like strains have been recovered from human and animal feces, which implies a commensal reservoir (*11**,**12*). Accordingly, we assessed our H18 EAEC isolates for membership in CGA.

A CGA-specific PCR protocol, which yields a 175-bp PCR product in strains that have 3 single-nucleotide polymorphisms within *fumC* (*13*), was applied to the Nigerian EAEC H18 isolates. Positive controls were 2 reference CGA cystitis isolates with UMN026 and SEQ102 (ATCC BAA-457) (*13*); negative controls were non-CGA cystitis isolate 2P9 (O15:K52:H1), UPEC isolates CFT073 (O6:K2:H1) and 536 (O6:K15:H31), and an H18 enterotoxigenic *E. coli* (ETEC) isolate from the Nigeria study (*14*). Of the 18 EAEC H18 isolates, 10 (including 8 from children with diarrhea) were positive, whereas 8 (and the H18 ETEC isolate) were negative. Lack of obvious familial or temporal clustering of patients from whom these strains were isolated suggests that the isolates are not likely to be directly linked through a single point source. Of 21 other EAEC strains from diverse non-African locales that were similarly screened, including 3 isolates bearing the H18 *fliC* allele (*1*), only Thai isolate 144-1 (H18-positive) (*1*) exhibited the CGA-specific *fumC* single-nucleotide polymorphisms. The 2 other non-Nigerian H18 EAEC (Peruvian O44:H18 isolate 042 and Thai O36:H18 isolate 44-1) (*1*) were negative.

To unequivocally assess clonal relationships, we subjected all H18 Nigerian EAEC isolates and the Thai isolate 144-1 to multilocus sequence typing (MLST) (*15*). Briefly, we sequenced designated internal regions of the *adk, fumC, gyrB, icd, mdh, purA,* and *recA* genes. Allele comparisons and sequence type (ST) assignments were done by using the open-source *E. coli* MLST database (http://web.mpiib-berlin.mpg.de/mlst/dbs/Ecoli). All 11 putative EAEC CGA isolates carried *fumC* allele 35, which has all 3 targets of the CGA single-nucleotide polymorphism screen (G270A, C271T, and C288T) (*13*). Of these isolates, 10 (9 Nigerian, 1 Thai) belonged to ST394, which shares 5 of 7 alleles with ST69 (the predominant ST of UPEC CGA; [*12*]) and, according to the e-BURST algorithm, is placed with ST69 in the same larger ST69 complex, indicative of a recent common ancestor (*15*). One Nigerian H18 EAEC isolate, strain E23, was assigned a new ST, ST432, because of its novel *purA* allele; however, ST432 shares alleles with ST394 at all 6 other loci and so also belongs with the ST69 complex. Of the 8 H18 isolates that were negative in the *fumC35* single-nucleotide polymorphism assay, 7 belonged to STs not previously described. Only 1 of these, strain E62 (ST471), shared 6 alleles with ST394 and 4 with ST69. Another strain, C16 (ST512), shared 5 alleles with ST69 and only 4 with ST394. Of the 8 *fumC35*-negative H18 isolates, 6 did not share 5 alleles with ST69 or ST394 and are therefore considered to be of a different clonal complex. Six *fumC35*-negative H18 isolates had 6 alleles in common and belonged to 1 of 3 STs: 31, 449, or 474. ST31 includes other EAEC in the MLST database (www.mlst.net). One EAEC H18 isolate shared no allele with any other EAEC isolate from this study (Table 2).

**Table 2:** Multilocus sequence types of CGA-associated *fumC* single-nucleotide polymorphism–positive strains*

Strain	Clinical condition, country	Allele profile							ST	ST complex
		*adk*	*fumC*	*gyrB*	*icd*	*mdh*	*purA*	*recA*		
C08	Diarrhea, Nigeria	21	35	61	52	5	5	4	394	69
C14	Diarrhea, Nigeria	21	35	61	52	5	5	4	394	69
E23	Diarrhea, Nigeria	21	35	61	52	5	72	4	432	69
E30	Diarrhea, Nigeria	21	35	61	52	5	5	4	394	69
G10	Diarrhea, Nigeria	21	35	61	52	5	5	4	394	69
G17a	Diarrhea, Nigeria	21	35	61	52	5	5	4	394	69
G59	Diarrhea, Nigeria	21	35	61	52	5	5	4	394	69
G67b	Diarrhea, Nigeria	21	35	61	52	5	5	4	394	69
E64	Healthy, Nigeria	21	35	61	52	5	5	4	394	69
G108	Healthy, Nigeria	21	35	61	52	5	5	4	394	69
144-1	Diarrhea, Thailand	21	35	61	52	5	5	4	394	69
SEQ102†	UTI (CGA), USA	21	35	27	6	5	5	4	69	69
C16	Healthy, Nigeria	21	22	2	6	5	5	4	512	Unassigned
G55	Healthy, Nigeria	6	4	33	1	20	12	7	423	Unassigned
E56	Healthy, Nigeria	18	22	17	6	5	5	4	31	31
E62	Healthy, Nigeria	21	125	61	52	5	5	4	471	Unassigned
E68	Healthy, Nigeria	18	22	94	6	5	5	4	449	Unassigned
G103	Healthy, Nigeria	18	22	94	6	5	5	4	449	Unassigned
G121a	Healthy, Nigeria	18	22	1	6	5	5	4	474	Unassigned
G149	Healthy, Nigeria	18	22	1	6	5	5	4	474	Unassigned

*CGA, clonal group A; ST, sequence type; UTI, urinary tract infection. †Data from reference (*14*). Data for all other isolates are from this study.

Although iron-utilization genes *chuA* and *iucA* are present in all EAEC and UPEC ST69 complex strains, the EAEC virulence plasmid markers *aggR*, *aap,* and CVD432 (*aat*) were found in all 11 EAEC ST394/432 isolates but not in reference UPEC CGA (ST69) isolates (Table 1). EAEC ST394/432 strains and UPEC ST69 (CGA) strains appear to represent diverging lineages of common ancestry, which are adapting to separate niches. Escobar-Paramo (*14*) proposed that certain *E. coli* backgrounds appear to be more likely to acquire virulence genes. Our findings suggest that the ST69 complex progenitor, from which CGA UPEC and ST394 EAEC are derived, may have had a propensity to acquire virulence genes as well as antimicrobial resistance elements, thereby generating at least 2 clonal groups pathogenic for humans, with several nonoverlapping, horizontally acquired virulence factors.

CGA-like strains recently isolated from animal feces and food samples have been proposed by Ramchandani et al. (*11*) as possible reservoirs for UPEC CGA strains. However, although they found similar resistance patterns and serotypes among animal CGA isolates as among humans, they did not find typical UPEC-associated virulence gene profiles (*11*). Tartof et al. (*12*) have subsequently shown that CGA-like strains from animal or environmental sources do not belong to ST69 (as do most human UPEC CGA isolates) but that they are part of the ST69 complex, particularly the ST394 type, which corresponds to the EAEC H18 clonal group we describe. Nonhuman ST394 isolates could possibly represent CGA-like EAEC and point to potential nonhuman reservoirs of EAEC, which remain to be identified.

## Conclusions

ST69 and ST394 appear to represent successful, genetically related lineages; isolates belonging to both are commonly resistant to ampicillin, chloramphenicol, streptomycin, sulfonamides, tetracycline, and trimethoprim. Widespread use of trimethoprim-sulfamethoxazole has been proposed as a reason for the emergence and spread of UPEC CGA (*8*). This combination, as well as other drugs to which ST394 strains are typically resistant, is commonly used (and misused) in Nigeria and other developing countries and could provide selective pressure for EAEC ST394/432. Although our numbers were too small to significantly associate ST394/432 strains with disease, 8 of 10 of these isolates were from children with diarrhea. Our study has unveiled what we believe to be a previously unrecognized EAEC clonal group. The *fumC* single-nucleotide polymorphism method, proposed for identifying UPEC CGA, could be useful for assessing its distribution.

## Supplementary Material

Appendix Table Flagellin types of EAEC strains isolated from Nigerian children identified by PCR-RFLP*
