# Supplementary material for: Enteroaggregative Escherichia coli Related to Uropathogenic Clonal Group A
Source: Emerg Infect Dis. 2007 May;13(5):757–60. doi: 10.3201/eid1305.061057 (PMC2738470; doi:10.3201/eid1305.061057)
Supplement: Appendix Table — Flagellin types of EAEC strains isolated from Nigerian children identified by PCR-RFLP* [file 06-1057_appT-s1.pdf]

Appendix Table. Flagellin types of EAEC strains isolated from Nigerian children identified by PCR-RFLP*

H-type†	Control strains‡	RFLP pattern§	EAEC isolates from children with diarrhea (%), n = 73	EAEC isolates from healthy controls (%), n = 58	Total (%), n = 131
H2	253-1 (O3:H2), 6-1(OR:H2)	D	3 (4)	1 (2)	4 (3.0)
H4	G02a (ONT:H4)	O	2 (3)	0 (0)	2 (1.5)
H6	EPEC E2348/69 (O127:H6)	K	1 (1)	0 (0)	1 (0.8)
H7 (no. 1)	EHEC EDL933 (O157:H7)	S	6 (8)	1 (2)	7 (5.3)
H7 (no. 2)	O128:H7	B	1 (1)	2 (4)	3 (2.3)
H11	C04 (O86:H11)	P	5 (7)	0 (0)	5 (3.8)
H18	042 (O44:H18), 44-1 (O36:H18), C08 (O86:H18)	A	10 (14)	8 (14)	18 (13.7)
H21	278-1 (O125ac:H21)	C	3 (4)	7 (12)	10 (7.6)
H40	G30a (O4:H40)	Y	2 (3)	0 (0)	2 (1.5)
H45	EPEC TWT 5350 (O157:H45)	H	5 (7)	1 (2)	6 (4.9)
ND	Not represented in the reference strain collection¶	J-AD (21 RFLPs)	20 (27)	27 (47)	47 (35.9)
H34, ND	EPEC 106 (H34)	No amplicon (not typable)	15 (21)	11(19)	26(19.5)
H16, H53	435-1 (O33:H16), 501-1 (OR:H53)	Patterns not seen in test strains	0 (0)	0 (0)	0 (0)

*EAEC, enteroaggregative *Escherichia coli*; RFLP, restriction fragment length polymorphism; EPEC, enteropathogenic *E. coli*, EHEC, enterohemorrhagic *E. coli*; ND, not determined.

†Determined by standard methods at a reference typing center.

‡Strains other than EPEC and EHEC were EAEC.

§Each RFLP was arbitrarily assigned a unique alphabetical identifier.

¶≤3 isolates showed each pattern.
